# *Propionibacterium acnes* Pyogenic Liver Abscess and Pylephlebitis

**DOI:** 10.1155/2011/536167

**Published:** 2011-10-09

**Authors:** Arash Poursina, Sharon Weissman

**Affiliations:** Department of Infectious Diseases, University of South Carolina, 2 Medical Park, Suite 205, Columbia, SC 29203, USA

## Abstract

Reported is an unusual case of pyogenic liver abscess and septic thrombophlebitis of the portal vein in a 44-year-old male caused by *Propionibacterium acnes* successfully managed with a combination of percutaneous drainage and antimicrobial therapy. To the best of our knowledge, this is the first report of this bacterium isolated in pure culture as the sole etiologic organism of pyogenic liver abscess and pylephlebitis in an adult patient.

## 1. Introduction

Pyogenic liver abscess is an infrequent infection with an incidence of 2.3–3.6 per 100,000 population [1, 2]. Although the incidence of pyogenic liver abscess appears to be increasing morbidity and mortality due to this condition has declined due to improved diagnostic and treatment modalities [3]. Pyogenic liver abscess can be a significant source of morbidity and mortality particularly if accompanied by septic thrombophlebitis of the portal system.

Although the source of the pyogenic liver abscess may be cryptogenic, the majority of cases result from biliary tract disease or spread followed by trauma and colonic disease [4, 5]. Enteric gram-negative bacilli are the most common organism involved, followed by streptococci species and anaerobic organism. Mixed infections are not uncommon [4, 5]. Septic thrombophlebitis has been reported to be the source of infection in thirteen percent of the cases [6]. Septic thrombophlebitis of the portal vein typically occurs in the setting of suppurative infection in the regions drained by the tributaries of the portal vein which includes the abdominal part of the gastrointestinal tract except the lower part of rectum [7].

We describe an unusual case of pyogenic liver abscess caused by *Propionibacterium acnes* with associated septic thrombophlebitis of the portal vein. 

## 2. Case Report

**Figure 1:**
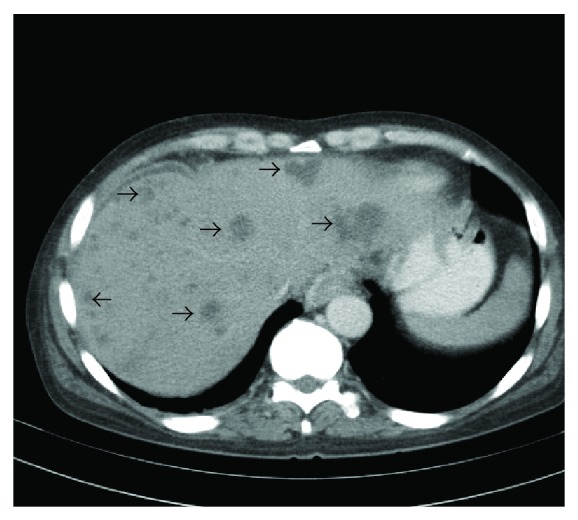
CT scan of the abdomen highly suspicious for multiple hepatic metastases.

**Figure 2:**
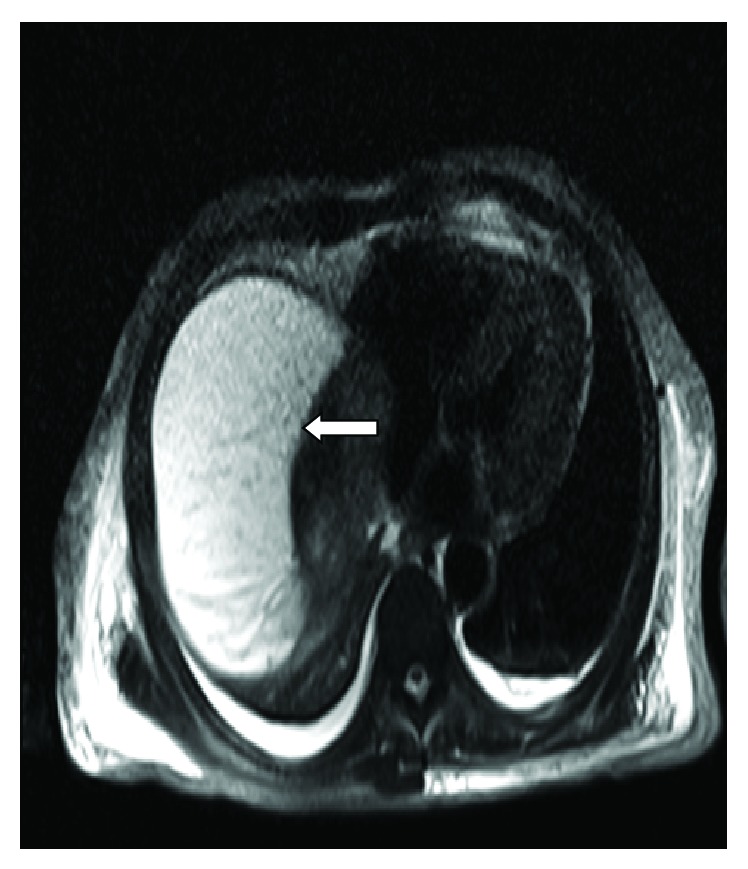
MRI of the abdomen with a large subcapsular abscess.

**Figure 3:**
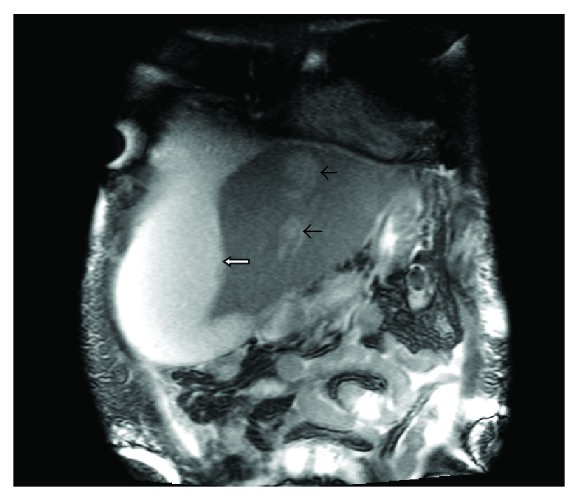
MRI of the abdomen with a large subcapsular abscess and coalescence of hepatic lesions into larger abscesses.

A 44-year-old male with a history of alcohol abuse presented with a one-week history of lethargy, confusion, stupor, abdominal pain, and jaundice. He had been seen in the emergency facility on several occasions during the preceding week. He was noted to have WBC count of 44,000 cell/*μ*L. Initial computed tomography scan of the abdomen showed a suspected pancreatic mass, and multiple liver lesions were felt to be of metastatic origin. He was referred to our facility for further evaluation. On arrival at our facility, he was noted to have a temperature of 100.7 F and a heart rate of 125/minute. On examination, he was jaundiced, stuporous, and confused. His abdomen was distended and diffusely tender. He had grossly edematous lower extremities. His laboratory blood work included a WBC 26.2 × 10^3^/*μ*L with 80% PMN, hemoglobin 9.8 g/dL, hematocrit 26.9%, and platelet count of 297 × 10^3^/*μ*L. His liver enzymes were elevated with AST 422 U/L, ALT 106 U/L, and Alkaline phosphatase 373 U/L. His plasma ammonia was 56 UMOL/L. His initial blood cultures, screening for viral hepatitis and alpha fetoprotein and CEA were all unrevealing. A repeat CT scan was reported to be strongly favoring extensive hepatic metastasis with a possible primary in the head of the pancreas (Figure 1). He underwent a CT-guided percutaneous biopsy. Histology revealed severe acute inflammatory with fibropurulent debris and no evidence of malignancy. A magnetic resonance imaging of his liver later showed rapid evolution of a large subcapsular abscess in the right lobe (Figure 2) and coalescence of the predominant lesions in the left liver lobe (Figures 2 and 3). His pancreatic abnormality was consistent with chronic pancreatitis. Doppler ultrasound of his portal vein was consistent with complete thrombosis. Computed-tomographic-guided percutaneous drainage of the large subcapsular abscess was performed under aseptic conditions. While awaiting the culture results, he was started on ceftriaxone and metronidazole. He was also started on coumadin for the management of his pylephlebitis. The percutaneous drainage gram stain was positive for predominantly intracellular gram-positive bacilli that could not be successfully grown in culture. His antibiotics were changed to imipenem and trimethoprim/sulfamethoxazole. An accidental dislodgement of his drain and the enlargement of his abscesses led to a second computed-tomography-guided percutaneous drainage a week later and a similarly positive gram stain but this time the cultures grew *Propionibacterium acnes*. His antibiotic coverage was narrowed to amoxicillin/clavulanic acid at 875/125 mg every 8 hours. He improved with antibiotics and percutaneous drainage of the abscess with complete resolution of his lesions on subsequent imaging studies and improvement in his functional status and laboratory parameters. Unfortunately, our patient died due to an unrelated cause several months later.

## 3. Discussion

We report an unusual case of *Propionibacterium acnes* as the etiologic agent for hepatic pyogenic liver abscess with associated thrombosis of the portal vein. To the best of our knowledge, there have only been three other cases of *Propionibacterium acnes *associated liver abscess in two of the cases this organism was part of a polymicrobial infection. The first case reported was in a four-year-old girl who developed a liver abscess four weeks after abdominal trauma. *Propionibacterium acnes *was isolated from the abscess and blood cultures [8]. The second case was reported in a 48-year-old man who intraoperatively was discovered to have a toothpick within the abscess. His intraoperative cultures grew *Streptococcus viridians*, *Bacteroides melaninogenicus*, and *Propionibacterium *[9]. The last case was reported in a 52-year-old woman with a mixed bacterial liver abscess. Percutaneous cultures grew *Actinomyces Israeli*, *Capnocytophaga spp*., and *Propionibacterium acnes. *She had an intrauterine device in place. This was presumptively the source of the infection [10]. 

*Propionibacterium acnes* is a commensal of human skin implicated in acne vulgaris. *P. acnes* has been implicated in cases of osteomyelitis of the spine, the skull, joints, and CNS infections and abscess formations often as the result of surgical interventions and postsurgical complications [11–13]. It is often associated with the presence of foreign bodies such as ventricular peritoneal shunts, trauma, malignancy, and immunodeficient conditions such as diabetes and immunosuppressive therapies [13].

This current case is different than the previous reports as *P. acnes* was isolated as the sole organism; there was not a foreign body involved or an antecedent trauma. Also, the current case was complicated by portal vein thrombosis. We postulate that septic thrombophlebitis of the portal vein was the source of the infection, although it is possible that *Propionibacterium acnes *was introduced iatrogenically during the initial needle aspiration. Arguments against an iatrogenic source in this case include (1) the multiple lesions seen on earlier imaging studies predated any invasive procedure, (2) the same lesions appeared to have coalesced to form larger abscesses and not all of them had been biopsied, (3) the presence of pylephlebitis as the source of the infective emboli in the liver prior to the percutaneous biopsies, and (4) relative impaired immunity of our patient due to his extensive history of alcohol abuse. 

This case serves as a reminder that, although rare, *Propionibacterium acnes* may be a cause of liver abscess. *P. acnes* may be an under recognized etiology due to difficulties in isolating the organism as it requires anaerobic media and a longer duration of incubation. The correct identification of this organism can help in a more directed narrow spectrum antibiotic management. Lastly, adequate aseptic precautions must be adhered to during percutaneous procedures to prevent introduction of skin commensals into deeper structures.
